# Long-run real-time PCR analysis of repetitive nuclear elements as a novel tool for DNA damage quantification in single cells: an approach validated on mouse oocytes and fibroblasts

**DOI:** 10.1007/s13353-023-00817-0

**Published:** 2023-12-18

**Authors:** Katarzyna Kotarska, Łukasz Gąsior, Joanna Rudnicka, Zbigniew Polański

**Affiliations:** 1 Laboratory of Genetics and Evolution, Institute of Zoology and Biomedical Research, Department of Biology, Jagiellonian University, Kraków, Poland; 2 Laboratory of Neurobiology of Trace Elements, Department of Neurobiology, Institute of Pharmacology, Polish Academy of Sciences, Kraków, Poland; 3 Malopolska Centre of Biotechnology, Jagiellonian University, Kraków, Poland; 4 Doctoral School of Exact and Natural Sciences, Jagiellonian University, Krakow, Poland

**Keywords:** DNA lesions, L1 elements, LORD-Q method, Single-cell genome, qPCR

## Abstract

Since DNA damage is of great importance in various biological processes, its rate is frequently assessed both in research studies and in medical diagnostics. The most precise methods of quantifying DNA damage are based on real-time PCR. However, in the conventional version, they require a large amount of genetic material and therefore their usefulness is limited to multicellular samples. Here, we present a novel approach to long-run real-time PCR-based DNA-damage quantification (L1-LORD-Q), which consists in amplification of long interspersed nuclear elements (L1) and allows for analysis of single-cell genomes. The L1-LORD-Q was compared with alternative methods of measuring DNA breaks (Bioanalyzer system, γ-H2AX foci staining), which confirmed its accuracy. Furthermore, it was demonstrated that the L1-LORD-Q is sensitive enough to distinguish between different levels of UV-induced DNA damage. The method was validated on mouse oocytes and fibroblasts, but the general idea is universal and can be applied to various types of cells and species.

**Supplementary Information:**

The online version contains supplementary material available at 10.1007/s13353-023-00817-0.

## Introduction

Evaluation of DNA damage in individual cells is a significant part of various research studies focused on such biological processes as cell division, cell death/survival, gamete maturation, and embryo development (Griffiths et al. 2018; Schier 2020; Xing et al. 2020). Also, reproductive medicine bases on single-cell analyses. They are used, among others, in the preimplantation diagnosis to assess the quality of gametes, embryos, and polar bodies. In these assessments, the degree of genome integrity is one of the important cell quality parameters (Wei et al. 2015). Although reliable technologies of single-cell transcriptomics, proteomics, epigenomics, and spatial omics have been introduced (Schier 2020), to our knowledge, there is no simple, sensitive, and fully quantitative single-cell method to evaluate DNA damage. NGS-based methods developed to map DNA breaks are not applicable for single cells since they require a high initial concentration of the targeted template (Baranello et al. 2014; Cao et al. 2019, 2020; Sriramachandran et al. 2020). Additionally, NGS becomes expensive and unnecessarily time-consuming when a small number of samples are to be analyzed (Schwarze et al. 2020). For the analysis of a limited number of high-concentration DNA samples, long-run real-time PCR-based DNA-damage quantification (LORD-Q) is a very competitive alternative. This method is based on two main assumptions: (1) lesions in DNA template block polymerase progression and reduce the level of amplification and (2) the probability that such lesions will appear in a long sequence is high and proportional to the general extent of DNA damage, but practically negligible and independent of DNA damage in the case of an extremely short sequence (Hunter et al. 2010; Furda et al. 2014; Lehle et al. 2014; Dannenmann et al. 2017). LORD-Q consists in the amplification of a long (3000–4000 bp) and an internally nested short fragment (40–70 bp) of the studied nuclear or mitochondrial genome and comparing the amounts of both PCR products (ΔCt value). The long fragment, which amplification decreases with increasing number of lesions in the DNA template, serves as an experimental probe. The short fragment, whose amplification does not depend on the degree of DNA integrity, serves as a normalization control. DNA damage in the examined samples (expressed in lesions per 10 kb) is calculated in relation to control samples which are assumed to be damage-free (Lehle et al. 2014; Dannenmann et al. 2017). LORD-Q is a very sensitive and accurate method that has been used successfully to quantify DNA lesions in various multicellular samples: cell lines and tissues (Lehle et al. 2014; Dannenmann et al. 2017; Zhu and Coffman 2017). The method could also be applied for single-cell analysis of mitochondrial DNA. Each cell contains many mitochondria which would provide enough input DNA template for PCR amplification. The problem arises, however, in the case of the nuclear genome—a single cell, depending on its type (germ/somatic) and cell cycle stage (before/after S phase), contains from one to four copies of distinctive coding sequence amplified in the classic LORD-Q assay, which is not sufficient to obtain a reliable PCR outcome. To overcome this problem, we developed a novel modification of the original LORD-Q method. Our strategy consists in replacing the amplification of one unique gene with the amplification of repetitive non-coding sequences LINE-1 (long interspersed nuclear element-1, L1). The size of L1 sequences is adequate for the demands of long-run PCR, and since repetitive elements are spread in thousands of copies along mammalian genomes (Waterston et al. 2002; Sookdeo et al. 2013), unlike the sequence of a unique gene, they provide an appropriate amount of input template for the reaction. By introducing L1 amplification to the LORD-Q assay, we created a new powerful tool for the measurement of DNA damage in singular cells. The novel approach has been validated mainly on oocytes for which evaluation of DNA quality is especially relevant. Additionally, we tested the modified method on fibroblasts to confirm that it can be applied to any type of cells. The primers used in our study are specific for mouse nuclear repetitive elements, but the general idea is universal and can be easily transferred to the analysis of other species, including people.

## Materials and methods

### Animals

Three- to four-month-old outbred OF1 mice (maintained in Institute of Zoology and Biomedical Research, Jagiellonian University, Kraków, Poland) were used for the experiments. The experiments were conducted at the Institute of Zoology and Biomedical Research of the Jagiellonian University according to the guidelines of the European Community Directive 86/609 and to the Polish Governmental Act on Animal Protection. The animals were maintained in a temperature- and light-controlled room (22 °C, 12-h light–dark cycle) and were provided with food and water ad libitum.

### Cells

Mouse fibroblasts (NIH/3T3 cell line; ATCC No. CRL-1658) in a concentration of 10^6^/ml, grown in high glucose Dulbecco’s Modified Eagle’s Medium (DMEM; Sigma Aldrich, St. Louis, MO, USA) supplemented with 10% fetal bovine serum (FBS; Gibco, Paisley, Scotland), penicillin (100 U/ml) and streptomycin (0.1 mg/ml) (Sigma Aldrich), were obtained from Department of Cell Biology; Faculty of Biochemistry, Biophysics and Biotechnology of Jagiellonian University, Kraków, Poland.

### DNA extraction from tail tissue and its fragmentation

DNA from the tip of a mouse tail was isolated with the GeneMATRIX Tissue DNA Purification Kit (EURx, Gdańsk, Poland) in accordance with the manufacturer’s instruction. The purity of the isolate (260/280 and 260/230 ratios) was verified on a Nanodrop 2000 spectrophotometer (Thermo Fisher Scientific, Waltham, MA, USA) and then divided into four equal parts. One part remained untreated (control DNA), and the three others were sheared in a Bioruptor Pico (Diagenode, Denville, NJ, USA) using 1, 2, or 3 sonication cycles (5/30 s on/off). The integrity of the control DNA and fragmentation of the sonicated DNA were confirmed by agarose gel electrophoresis (Figure S1). The fragmented DNA samples were measured on the Qubit Fluorometer (Invitrogen, Waltham, MA, USA) using the Qubit 1X dsDNA HS Assay Kit (Invitrogen), then diluted to a concentration of 1 ng/μl, and analyzed with a 2100 Bioanalyzer system (Agilent, Santa Clara, CA, USA) to determine the average length of the generated fragments. The same samples (diluted to a concentration of 0.5 ng/μl) were reanalyzed with the LORD-Q assay using the same procedure as for single cells.

### Collection of oocytes

Oocyte isolation and further handling were performed under stereomicroscope using mouth pipette as previously described (e.g. Polanski et al. 2005). Female mice were killed by cervical dislocation, and their ovaries were dissected and placed in M2 culture medium supplemented with 150 μg/ml dbcAMP (Sigma-Aldrich) to prevent meiosis resumption. The oocytes were then released from the ovaries by puncturing the follicles and collected in a drop of M2 medium with dbcAMP under mineral oil. Only oocytes at the first meiotic prophase stage (with visible germinal vesicle, GV) were used for further procedures.

### Induction of DNA damage in oocytes and fibroblasts

GV oocytes in a 100 μl M2 drop or DMEM suspension of fibroblasts diluted 10X with M2 medium (100 μl) were exposed to ultraviolet C (UV-C) radiation (wavelength 254 nm) at a dose of 15, 30, 60, or 120 mJ/cm^2^ in a UV crosslinker (UVP HL-2000 HybriLinker, UVP, Upland, CA, USA). After irradiation, single fibroblasts were individually collected in the buffer for DNA extraction, while single oocytes were collected either for DNA extraction or for immunofluorescent staining.

### Immunofluorescent staining of oocytes and confocal microscopy

The oocytes were briefly washed in PBS and then fixed in 4% paraformaldehyde in PBS for 15 min at room temperature. After washing in PBS (3× for 10 min), the oocytes were permeabilized for 15 min in 0.1% Triton X 100 in PBS and incubated in 10% fetal bovine serum (FBS) at room temperature. Then, the oocytes were incubated overnight at 4 °C with the primary anti-γ-H2AX antibody (Cell Signaling Technology, Danvers, MA, USA, 9718) diluted 1 : 100 in 10% FBS. After washing in PBS (3× for 10 min), the oocytes were incubated for 1 h at room temperature with secondary Cy3-conjugated goat anti-rabbit antibody (Abcam, Cambridge, UK, ab6939), used at a dilution of 1 : 500 in 10% FBS. For nuclear DNA visualization, after washing in PBS (3× for 10 min), the oocytes were incubated for 15 min in HCS NuclearMask™ (Thermo Fisher Scientific), then washed in PBS for 10 minutes, and mounted in 1 μl PBS droplets using a Vaseline layer between a slide and a cover glass to preserve the oocyte three-dimensional structure. The mounted specimens were analyzed with the ZEISS LSM 880 Confocal Laser Scanning Microscope (Oberkochen, Germany) using the 20× Zeiss Plan-Apochromat Infinitive corrected objective with a numerical aperture of 0.8. Fluorescence signal intensities from irradiated oocytes were measured using ImageJ software and normalized to control (non-irradiated oocytes).

### DNA extraction from oocytes and fibroblasts

Single control or irradiated oocytes/fibroblasts were placed individually, with the help of mouth pipette, in 0.2 ml tubes containing 40 μl of Taq Buffer with KCl (Thermo Fisher Scientific) and proteinase K (200 μg/ml) (Sigma-Aldrich). Cells in the lysis buffer were heated at 55 °C for 30 min followed by heating at 95 °C for 10 min for proteinase K deactivation. The lysates were then stored at −20 °C until the real-time PCR analysis.

### Preparation of the standard of L1 copy number

Long fragment (3494 bp) of L1 was amplified with PCR using Takara LA Taq DNA Polymerase Hot-Start Version (Shiga, Japan) and primers designed for the L1-LORD-Q assay at a concentration of 200 nM each. The reaction was carried out in a volume of 50 μl on 250 ng of input DNA isolated from mouse tail tissue. The cycling conditions were as follows: (1) 1 min at 94 °C; (2) 35 cycles of 10 s at 98 °C, 30 s at 60 °C, and 3 min at 68 °C; (3) 10 min at 68 °C. The PCR product was separated from other components of the reaction by agarose gel electrophoresis and then extracted from agarose using the Gel-Out kit (A&A, Gdańsk, Poland). The purified fragment of L1 was inserted into the pGEM-T Easy Vector (Promega, Madison, WI, USA) and cloned in JM106 *E. coli* cells according to the manufacturer’s instructions. Vectors were extracted from bacteria culture with the Plasmid Mini kit (A&A), and their concentration was measured in Nanodrop. The obtained plasmid was verified to have the L1 sequence by PCR, restriction enzyme digestion, and electrophoresis. After successful verification, the plasmid was diluted to a concentration of 10^6^ copies per 40 μl and used as a standard in real-time PCR assays.

### L1-LORD-Q assay

Primers designed for single-cell real-time PCRs amplify long (3494 bp) and internally nested short fragment (66 bp) of a conserved region of the L1 ORF2 sequence. The reverse primer is the same for both reactions, while the forward primers determine the length of the PCR product (Table 1). The primers were designed based on L1Base 2 (http://l1base.charite.de) containing sequences of putatively active L1 insertions residing in human and rodent genomes (Penzkofer et al. 2017). Primer specificity was confirmed by agarose gel electrophoresis and melt curve analysis (Figure S2). Taq polymerase and standard SYBR Green I dye were used for the amplification and real-time detection of the short L1 sequence. In order to ensure proper amplification of the long sequence, high-fidelity and rapid PrimeSTAR GXL polymerase was employed in combination with ResoLight dye, which is less inhibitory to DNA polymerase during long-run PCR.

**Table 1:** Primers used for the L1-LORD-Q assay and mean PCR efficiencies (± SD)

L1 fragment	Short	Long
Size [bp]	66	3494
Forward primer sequence 5′–3′	GGCGAGGATGTGGAGAAAG	CAGCCACAAGAACAGAATGC
Reverse primer sequence 5′–3′	GTGGTTGTACAAGCCTGCAA	
PCR efficiency	1.98 (± 0.08)	1.82 (± 0.06)

The reaction mixture for the short fragment included 2 μl of DNA template, PowerUp SybrGreen Master Mix (Applied Biosystems, USA), and 200 nM of each primer in a total volume of 10 μl per well. Real-time analysis was performed on the QuantStudio 5 Real-Time PCR System (Applied Biosystems, Waltham, MA, USA). The cycling conditions were as follows: (1) 2 min at 50 °C, (2) 2 min at 95 °C, (3) 40 cycles of 15 s at 95 °C, and 1 min at 60 °C. Next, a melt curve was drawn to ensure that there was no unspecific amplification and/or primer–dimer formation. The reaction mixture for the long fragment included 2 μl of DNA template, 0.4 μl of PrimeSTAR GXL Polymerase (Takara), PrimeSTAR GXL Buffer (Takara), dNTP mix (200 μM each) (Takara), primers (200 nM each), and 0.05 of ResoLight Dye (Roche, Basel, Switzerland) in a total volume of 10 μl per well. Real-time analysis was carried out on the LightCycler 96 System (Roche). The cycling conditions were as follows: (1) 1 min at 98 °C, (2) 40 cycles of 10 s at 98 °C, 15 s at 60 °C, and 1 35 s at 68 °C. Next, a melt curve was drawn to ensure that there was no unspecific amplification and/or primer–dimer formation.

In each 96-well PCR plate, apart from samples, serial two-fold dilutions of the copy number standard were amplified (7 points from 5 × 10^5^ to 7.8 × 10^3^copies). On the basis of standard curves, the efficiency of the reactions in the subsequent plates was calculated (Figure S3). The standard curves created for the amplification of the short fragment were additionally used to assess the number of L1 sequences amplified in a single-cell genome during the assay. All DNA samples were run in triplicate. Mean Ct values derived from real-time PCR runs together with amplification efficiencies were used to calculate the number of DNA lesions per 10 kb in the examined material. For the above calculations, the equation introduced by Lehle et al. (2014) was used:

$$\frac{\textrm{lesions}}{10\ \textrm{kb}}=10\ 000\times \left[{\left(\frac{{E_L}^{\textrm{CtL}\left(\textrm{sample}\right)}\times {E_S}^{-\textrm{CtS}\left(\textrm{sample}\right)}}{{\left[\begin{array}{c}\left({E_L}^{\textrm{CtL}\left(\textrm{control}\ 1\right)}\times {E_S}^{-\textrm{CtS}\left(\textrm{control}\ 1\right)}\right)\times \\ {}\dots \times \left({E_L}^{\textrm{CtL}\left(\textrm{control}\ n\right)}\times {E_S}^{-\textrm{CtS}\left(\textrm{control}\ n\right)}\right)\end{array}\right]}^{1/n}}\right)}^{1/a}-1\right]$$

*E*_*L*_: amplification efficiency of the long fragment
*E*_*S*_: amplification efficiency of the short fragment
*Ct*_*L*_: threshold cycle of the long fragment
*Ct*_*S*_: threshold cycle of the short fragment
*a*: number of base pairs of the long fragment
*n*: number of control samples

### Statistics

All sets of data were checked for Gaussian distribution with the Shapiro–Wilk test. Since not all data appeared to be normally distributed, differences between groups were analyzed with the nonparametric Kruskal–Wallis test. *P* value below 0.05 was considered statistically significant.

## Results and discussion

**Fig. 1:**
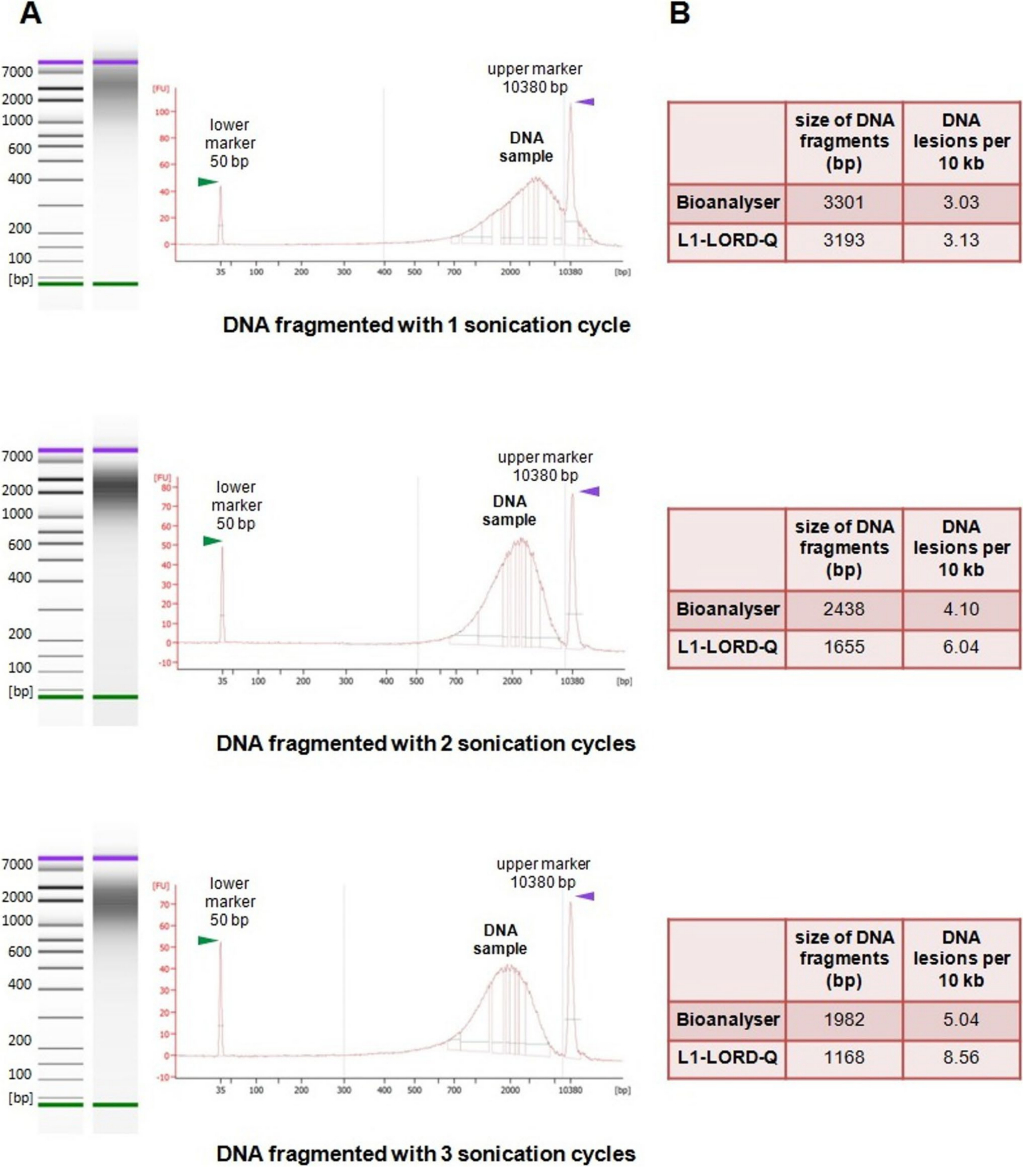
The L1-LORD-Q assay accurately reproduced different levels of fragmentation in DNA samples treated with 1, 2, or 3 sonication cycles. **A** Automated electrophoresis and size profiles of differently fragmented DNA samples generated by the Agilent 2100 Bioanalyzer system. **B** Average size of DNA fragments and the corresponding number of DNA lesions measured in the same samples by the Bioanalyzer and by the modified LORD-Q assay

In the first stage of our study, we explored whether amplification of L1 elements in the LORD-Q assay distinguishes different degrees of DNA fragmentation and shows the actual state of the analyzed templates. From freshly isolated mouse DNA, we prepared samples fragmented with 1, 2, and 3 sonication cycles. High-resolution automated electrophoresis (Agilent Bioanalyzer system) allowed for precise evaluation of the average size of DNA fragments in subsequent samples. As expected, this size was the largest in the sample subjected to single sonication (3301 bp), medium in the sample subjected to double sonication (2438 bp), and the smallest in the sample received after triple sonication (1982 bp). The length of DNA fragments could be easily converted to the number of DNA breaks per 10 kb, which is the standard way of presenting results in LORD-Q. The conversion gave 3.03 breaks in the first sample, 4.10 breaks in the second sample, and 5.04 breaks in the third sample. Then, the same samples were used as templates in real-time PCRs amplifying short and long sequences of L1. The modified LORD-Q assay correctly reproduced the gradual increase in DNA fragmentation in samples subjected to 1, 2, and 3 sonication cycles, estimating the number of DNA lesions per 10 kb at 3.13, 6.04, and 8.56, respectively (Fig. 1). Comparing the results obtained for the same samples after automated electrophoresis and PCR, it can be concluded that the L1-LORD-Q shows more lesions than the Bioanalyzer system. Only in the case of the least fragmented sample, both methods gave the same estimations, possibly because some fragments in this sample were longer than 10,380 bp (upper marker) and escaped from the electrophoretic analysis. Polymerase-driven DNA duplication in PCR is inhibited not only by double-strand breaks but also by some other types of DNA lesions, such as thymine dimers, abasic sites, and 5-hydroxymethylcytosines (Lehle et al. 2014). These lesions, which can be detected by LORD-Q but do not influence electrophoresis, probably contribute to some discrepancy between the values assessed by PCR amplification and the Bioanalyzer. Nevertheless, the real-time PCRs showed accurate levels of DNA damage corresponding to the direct electrophoretic measurements. This proved that the DNA damage rate within L1 sequences is representative for the whole genome.

**Fig. 2:**
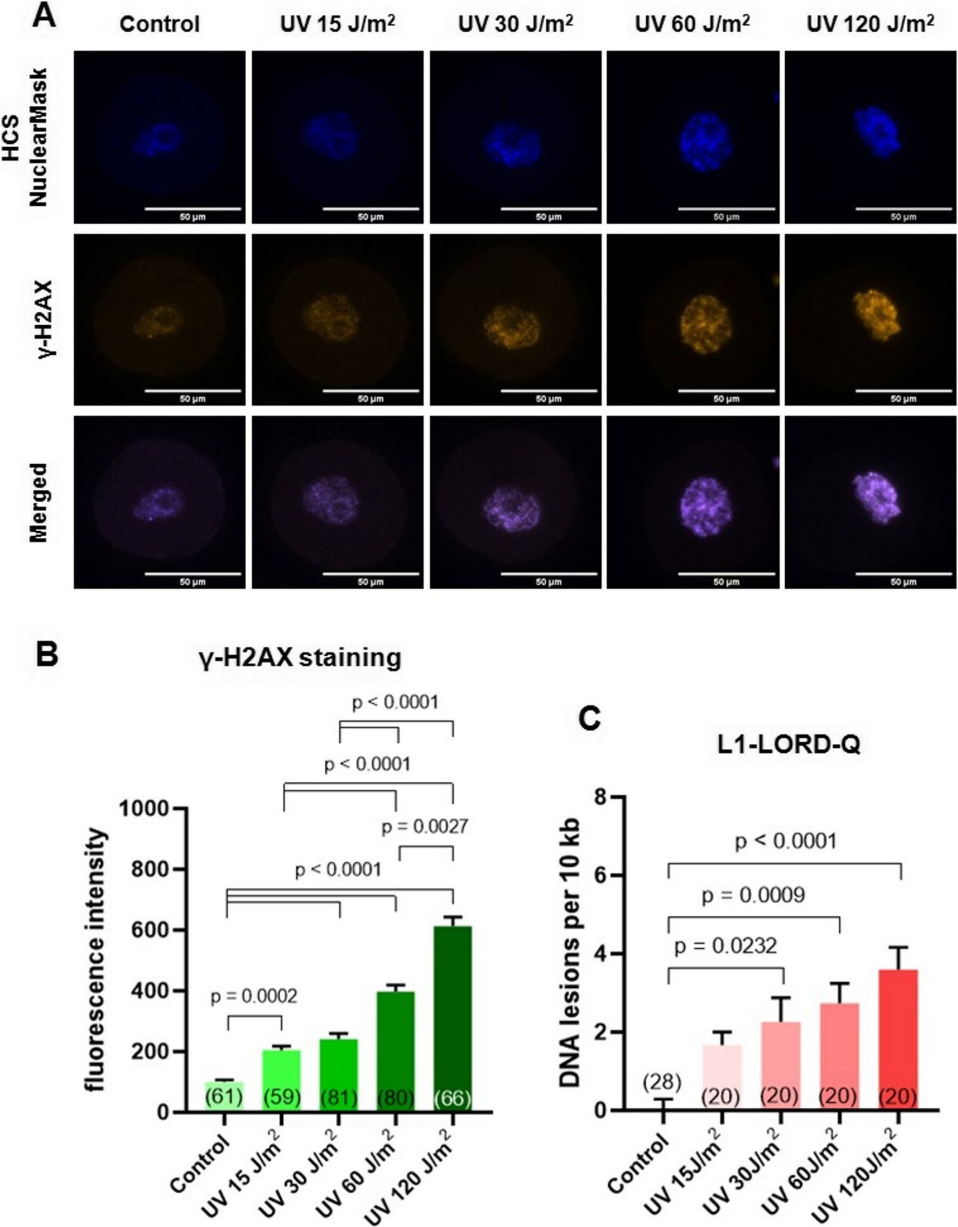
The L1-LORD-Q assay allows quantifying the number of DNA lesions in single oocytes treated with different doses of UV light and shows a level of damage comparable to the standard immunofluorescent method. **A** Exemplary control and UV-treated oocytes stained with antibody against γ-H2AX—a popular marker of DNA double strand breaks. **B** Level of DNA breaks in control and UV-treated oocytes assessed by immunofluorescent labeling of γ-H2AX foci (means ± SEM, in brackets: number of oocytes analyzed in each group). **C** Number of DNA lesions in control and UV-treated oocytes assessed by the modified LORD-Q assay (means ± SEM, in brackets: number of oocytes analyzed in each group)

Since the Bioanalyzer is not sensitive enough to analyze genomes of single cells, multicellular DNA was used to compare its measurements with the modified LORD-Q quantification. However, the next stages of our study were performed exclusively on individual cells. Our laboratory specializes in the biology of gametes and therefore the L1-LORD-Q assay was validated primarily on oocytes. We induced DNA damage in individual prophase oocytes using growing doses of UV-C light (15, 30, 60, and 120 J/m^2^) and then analyzed the genomes of irradiated gametes with immunofluorescent staining of γ-H2AX foci or with the L1-LORD-Q. The phosphorylation of Ser139 in the histone variant H2AX (γ-H2AX) is an early cellular response to the induction of DNA double-strand breaks (DSBs); hence, γ-H2AX foci labeling is considered a sensitive and reliable method of quantifying DSBs (Mah et al. 2010; Stringer et al. 2020). Our analysis of γ-H2AX foci revealed very low fluorescence in control oocytes, and significant, dose-dependent fluorescence increase in UV-treated oocytes, indicative of increasing numbers of DNA DSBs in subsequent experimental groups. The same pattern of gradual increase of DNA damage with increasing UV dose was obtained after analyzing oocytes with the L1-LORD-Q assay (Fig. 2). It shows that our PCR-based method is equally effective in detecting DNA breaks in oocytes as standard γ-H2AX staining, and in some aspects, we find it more attractive. The L1-LORD-Q can be automated. It is simpler to perform and less time-consuming than immunofluorescent analysis. PCR amplification detects not only DSBs but also a few other types of DNA damage (Lehle et al. 2014). It directly quantifies DNA lesions and can be applied just after their induction. In contrast, H2AX phosphorylation is an indirect marker of DNA breaks and can be measured only after a certain duration following exposure to the stressor because cells need time to respond to DSB induction in their genome (Stringer et al. 2020). Moreover, γ-H2AX foci staining is reliable when interphase cells are analyzed but becomes inaccurate when applied to highly condensed M-phase mitotic and meiotic chromatin. This is caused by technical problems with signal measurement and by the fact that the role of γ-H2AX in this case is not restricted to the response to DNA damage but exceeds to regulation of some cell cycle events (Turinetto and Giachino 2015). Finally, the big advantage of the L1-LORD-Q is that it gives the real value of the amount of DNA damage in the analyzed cell. This cannot be achieved using γ-H2AX labeling which only allows for comparisons of staining intensity between different cells to determine relative levels of DNA damage. Furthermore, statistical significance in staining intensity between cells does not necessarily translate in statistical significance in the real level of DNA damage, since the exact relationship between staining intensity and DNA damage rate is unknown.

**Fig. 3:**
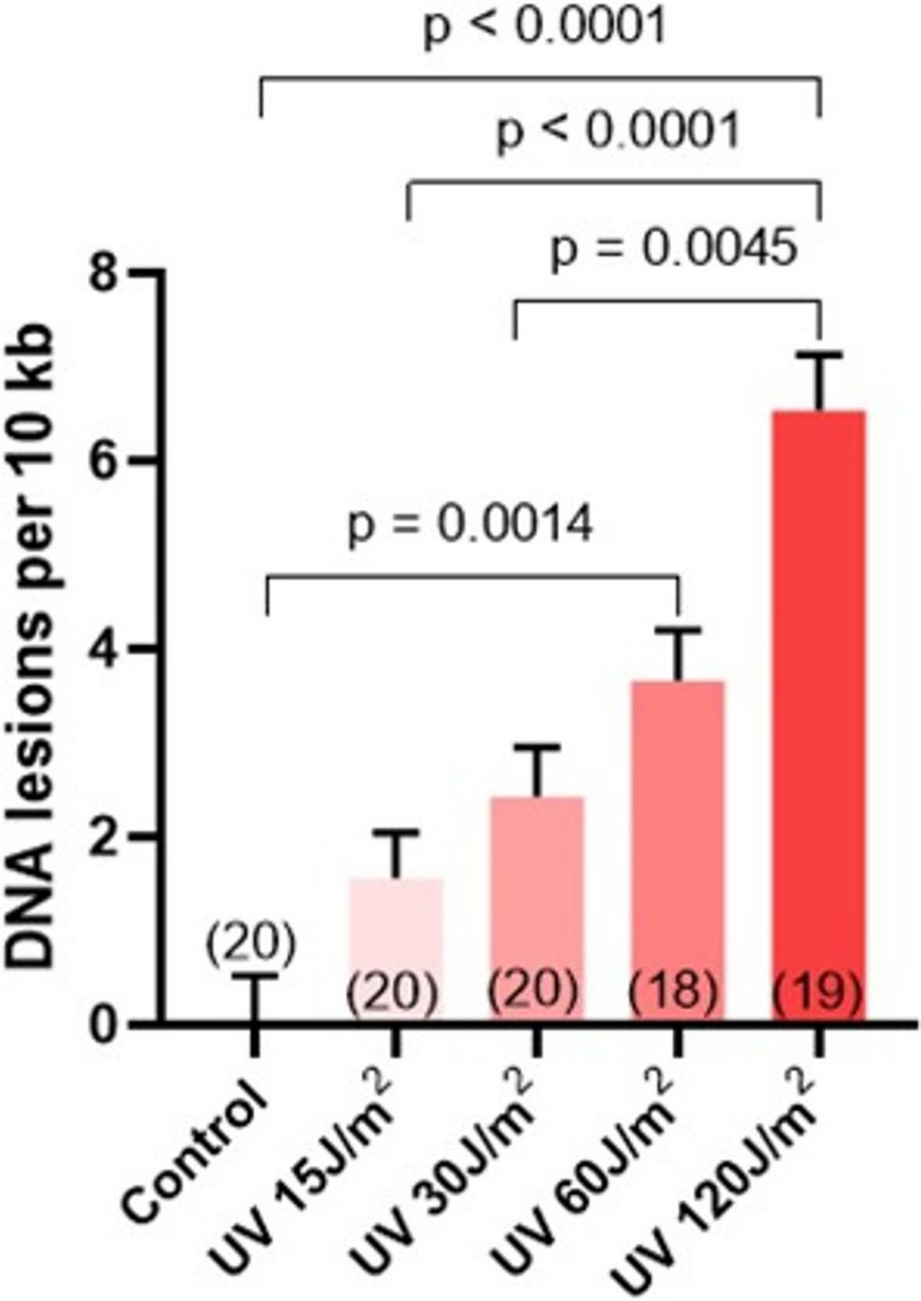
The L1-LORD-Q assay is universal and allows detecting DNA damage not only in oocytes but also in any other single cell. The figure shows the number of DNA lesions in single mouse fibroblasts treated with different doses of UV light and analyzed with the modified LORD-Q assay, analogically to oocytes (means ± SEM, in brackets: number of oocytes analyzed in each group)

After demonstrating that the L1-LORD-Q is an effective and accurate tool for quantifying DNA lesions in oocytes, we decided to verify our method on other types of cells. To this end, we irradiated individual mouse fibroblasts with different doses of UV-C light and then analyzed their genomes with real-time PCRs employing an identical procedure as for oocytes. Similar to the findings in oocytes, we obtained a comparable trend in the gradual increase of DNA damage with increasing UV dose (Fig. 3). The number of DNA lesions was slightly higher in fibroblasts than in prophase oocytes at the two highest UV doses, which could be associated with lower chromatin condensation of somatic cells and the resulting greater susceptibility to damage induction. Nevertheless, the L1-LORD-Q demonstrated the capability to accurately estimate the number of DNA lesions in fibroblasts, comparable to its efficacy in oocytes, thereby demonstrating the universal applicability of this approach across diverse cell types.

**Fig. 4:**
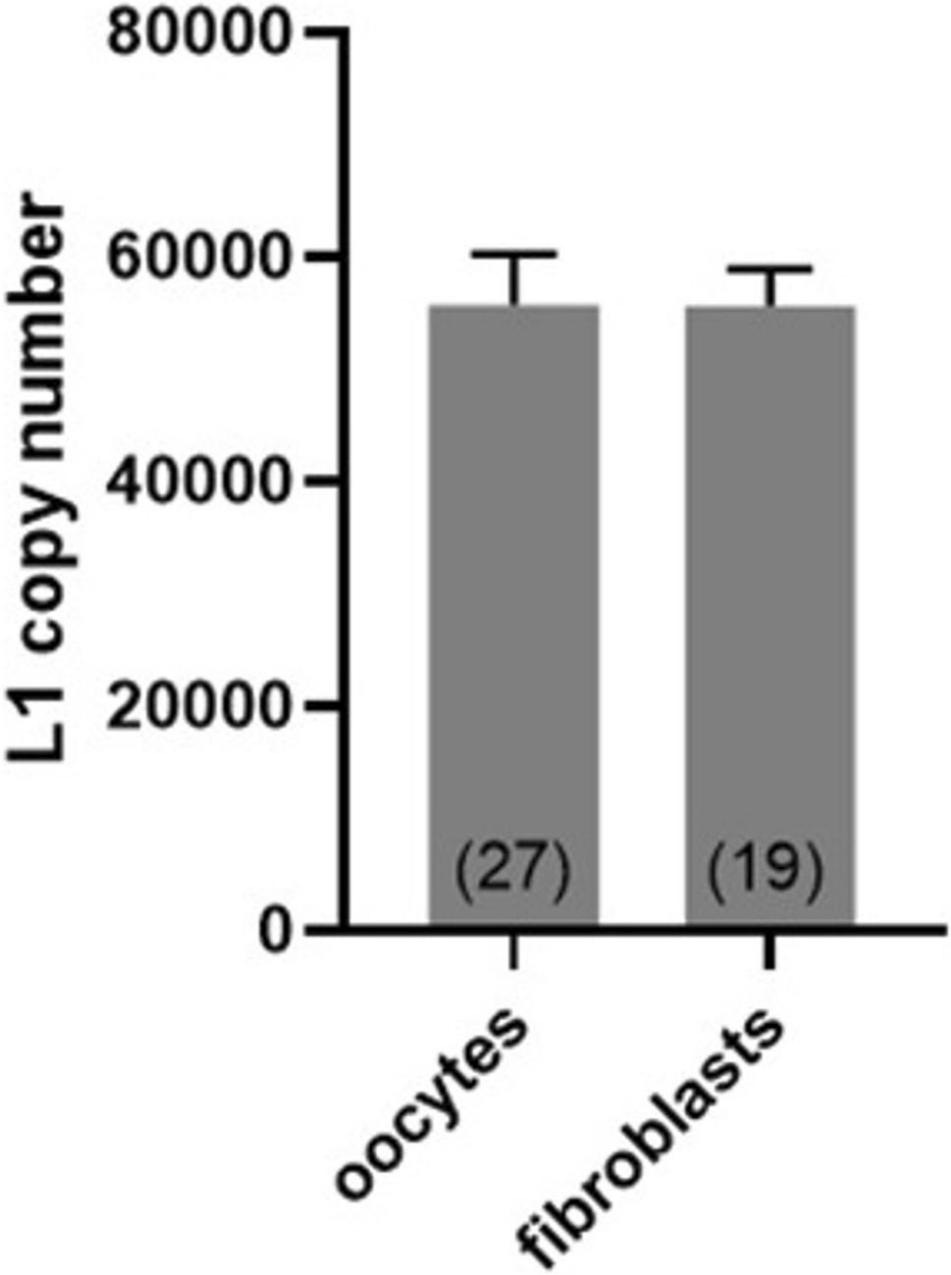
Number of L1 sequences in the genome of prophase I oocytes and fibroblasts, which are amplified during the L1-LORD-Q assay (means ± SEM). L1 copy numbers were assessed for amplification of a short fragment using a plasmid based L1 copy number standard curve

For each real-time PCR plate, we created a standard curve using serial dilutions of the L1 copy number standard. Such standard curves allowed not only to read the reaction efficiency but additionally to assess the number of L1 sequences in single-cell genomes that are amplified with the employed primers. This number appeared to be almost the same for control oocytes: 55,722.6 copies, and for control fibroblasts: 55,624.7 copies (Fig. 4). The assessment showed the power of our approach—over 55,000 sequences within one diploid genome are the template in the first PCR cycle. This enables a reliable analysis of single-cell DNA in contrary to the classical LORD-Q assay, in which one unique gene is amplified (Lehle et al. 2014; Dannenmann et al. 2017). Our standard curves were generated from serial dilutions of the L1 copy number standard. It should be emphasized, however, that in routine single-cell DNA damage quantification, the plasmid-based copy number standard is not indispensable. Serial dilutions of any DNA sample can be used to determine PCR efficiency (needed for the final calculation).

We strongly believe that the L1-LORD-Q is a powerful tool with broad applications, but simultaneously, we realize that, as all analytical methods, it has some limitations. Similarly to the previous PCR-based methods of DNA damage quantification, our approach detects only these types of lesions that inhibit polymerase progression. For instance, modifications of nitrogen bases typical for UV damage, such as 8-hydroxydeoxy-guanosine (8-OHdG), do not affect the efficiency of PCR amplification and thus cannot be recognized with any type of LORD-Q (Sikorsky et al. 2004). Moreover, the nondiscriminatory nature of LORD-Q cannot provide information about the type of DNA lesion. This would limit the usability of our assay when the experimental goal is to study exclusively one specific type of damage in a single-cell genome. Finally, our approach is based on the assumption that the L1 elements are evenly distributed in mammalian genomes and that all DNA fragments are equally prone to DNA damage, which is a simplification. In fact, L1s are not quite randomly distributed and occur primarily in the AT-rich and gene-poor regions (Graham and Boissinot 2006). Previous studies indicate, additionally, that coding regions are more prone to the formation of DNA lesions than transcriptionally inactive regions (Lu et al. 2004; Bouwman and Crosetto 2018). Theoretically, regions containing more L1s may be, for this reason, less sensitive to damage than euchromatic gene-rich parts of the genome. Moreover, the mechanisms of DNA lesion formation vary between stressors. For instance, some DNA damage agents are known to have a greater ability to introduce DNA breaks into the AT-rich noncoding regions, while others into the GC-rich coding regions (Woynarowski 2002). Taking into account all the above reservations, the whole-genome damage rates assessed with the L1-LORD-Q assay should be considered as approximate values. The approximate nature of the obtained values results additionally from the fact that the number of DNA lesions in the experimental samples is calculated in relation to the control samples, where DNA damage is assumed to be zero, although this is not entirely true. The formation of a small portion of DNA lesions is a physiological phenomenon occurring spontaneously in all cells as a consequence of their metabolism and interactions with the environment (Vilenchik and Knudson 2003; Martin 2008; Yousefzadeh et al. 2021).

Despite its limitations, the modified LORD-Q assay is a quick, low-cost, effective, and sensitive tool to assess the general rate of DNA lesions in single-cell genomes. The conducted experiments proved that it is capable of distinguishing with high accuracy between samples with varying degrees of DNA damage. We demonstrated that the results obtained with the L1-LORD-Q, expressed in lesions per 10 kb, are close to reality. They directly represent DNA damage rate in contrast to the fluorescence intensity obtained after popular γ-H2AX staining. Our novel approach is universal and can be applied to any cell type and to a variety of species due to the widespread presence of repetitive elements in animal and plant genomes. As other PCR-based methods, the L1-LORD-Q can be automated and used for routine DNA damage assessments, both in scientific research and in medical diagnostics. Finally, development and optimization of biotechnically efficient L1-LORD-Q-mediated approaches to identify, in a highly detectable manner, the diminished molecular quality of single fibroblasts and/or oocytes might provide an excellent tool largely applicable to the negative selection of nuclear donor somatic cells and/or nuclear recipient germ cells that seem to be genetically undesirable/unsuitable for a wide variety of modern assisted reproductive technologies (ARTs) in mice and other mammalian species. This variety of ARTs encompasses such innovative strategies of in vitro embryo production (IVP) as somatic cell nuclear transfer (SCNT)-based cloning combined or not combined with transgenesis (Song et al. 2020; Skrzyszowska and Samiec 2021; Wiater et al. 2021; Moradi-Hajidavaloo et al. 2023) and in vitro fertilization (IVF) by either gamete coincubation (Treleaven et al. 2021; Gorczyca et al. 2022; Montgomery et al. 2023) or intracytoplasmic sperm injection (ICSI) (Yin et al. 2021; Campos et al. 2023; Ramírez-Agámez et al. 2023).

### Supplementary information

ESM 1 **Supplementary figure S1.** Agarose gel electrophoresis of mouse intact DNA (control) and the same DNA, but fragmented with 1, 2, or 3 sonication cycles (F1, F2, and F3, respectively). **Supplementary figure S2.** Specificity of primers used for the amplification of L1 short and long fragments. **(A)** Agarose gel electrophoresis of short and long PCR products. **(B)** Melting curves of short and long PCR products (-ΔF/ΔT vs. temperature). **Supplementary figure S3.** Exemplary standard curves for L1 short and long fragments created after performing real-time PCR on two-fold serial dilutions of a plasmid-based standard. Quantities of consecutive points on the standard curve: 0.008, 0.016, 0.031, 0.062, 0.125, 0.25 and 0.5 × 10^6^ copy number. Standard curves were created for each PCR plate to determine reaction efficiency. (PDF 351 kb)
