# Supplementary material for: Long-run real-time PCR analysis of repetitive nuclear elements as a novel tool for DNA damage quantification in single cells: an approach validated on mouse oocytes and fibroblasts
Source: J Appl Genet. 2023 Dec 18;65(1):181–90. doi: 10.1007/s13353-023-00817-0 (PMC10789673; doi:10.1007/s13353-023-00817-0)
Supplement: Supplementary file 1 — Supplementary figure S1. Agarose gel electrophoresis of mouse intact DNA (control) and the same DNA, but fragmented with 1, 2, or 3 sonication cycles (F1, F2, and F3, respectively). Supplementary figure S2. Specificity of primers used for the amplification of L1 short and long fragments. (A) Agarose gel electrophoresis of short and long PCR products. (B) Melting curves of short and long PCR products (-ΔF/ΔT vs. temperature). Supplementary figure S3. Exemplary standard curves for L1 short and long fragments created after performing real-time PCR on two-fold serial dilutions of a plasmid-based standard. Quantities of consecutive points on the standard curve: 0.008, 0.016, 0.031, 0.062, 0.125, 0.25 and 0.5 × 10^6^ copy number. Standard curves were created for each PCR plate to determine reaction efficiency. (PDF 351 kb) [file 13353_2023_817_MOESM1_ESM.pdf]

Supplementary Figure S1

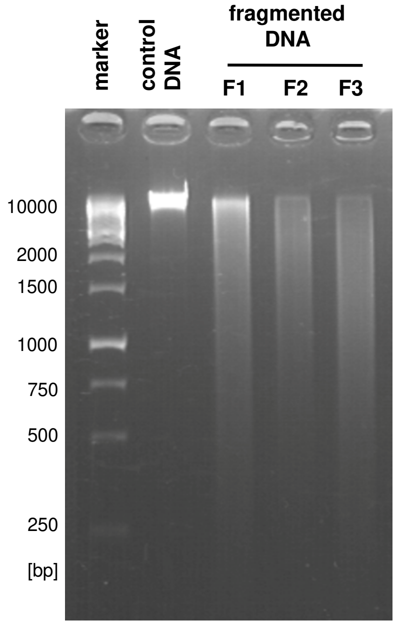

Supplementary Figure S2

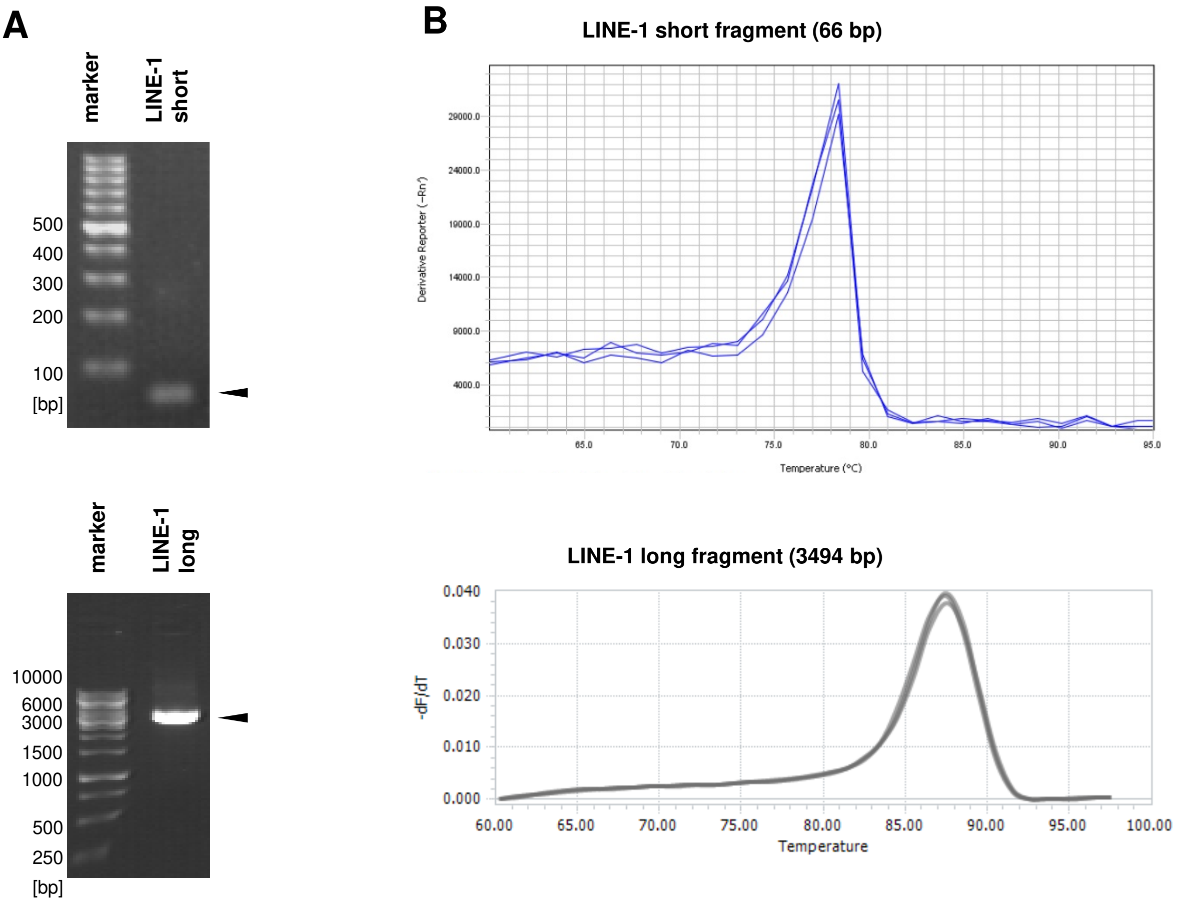

Supplementary Figure S3

LINE-1 short fragment

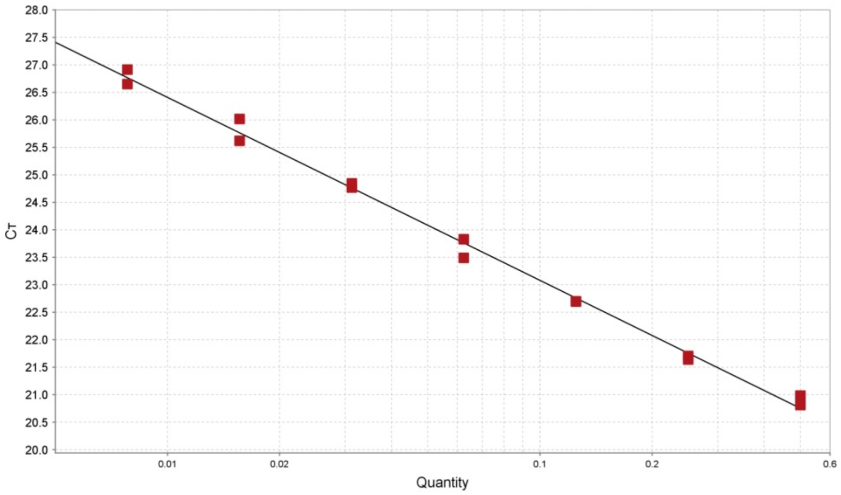

Efficiency: 1.99

LINE-1 long fragment

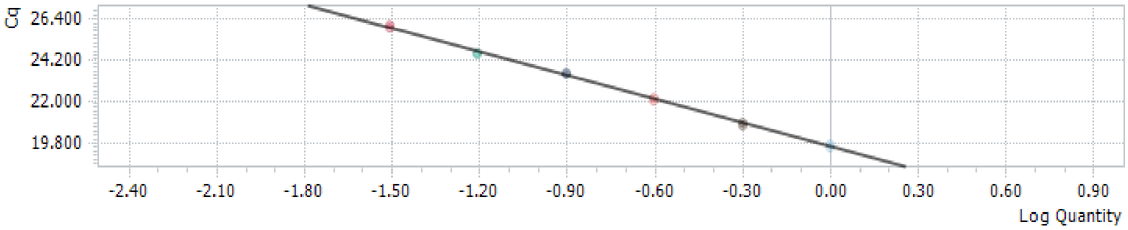

Efficiency: 1.74
